# A Pilot Investigation of the Hidden Communities Associated With *Dryas octopetala* L. (Rosaceae) in Svalbard Using DNA Metabarcoding

**DOI:** 10.1111/1758-2229.70223

**Published:** 2025-11-05

**Authors:** Micheline Carvalho‐Silva, Luiz H. Rosa, Vívian N. Gonçalves, Marcelo H. S. Ramada, Kauana Beppler de Souza, Gabrielle S. M. de Araújo, Fabyano A. C. Lopes, Peter Convey, Paulo E. A. S. Câmara

**Affiliations:** ^1^ Departamento de Botânica Universidade de Brasília Brasília Brazil; ^2^ Departamento de Microbiologia Universidade Federal de Minas Gerais Belo Horizonte Brazil; ^3^ Universidade Católica de Brasília Brasília Brazil; ^4^ Laboratório de Microbiologia Universidade Federal do Tocantins Porto Nacional Brazil; ^5^ British Antarctic Survey Cambridge UK; ^6^ Department of Zoology University of Johannesburg Johannesburg South Africa; ^7^ Millennium Institute—Biodiversity of Antarctic and Sub‐Antarctic Ecosystems (BASE) Santiago Chile; ^8^ School of Biosciences University of Birmingham Birmingham UK; ^9^ Pós Graduação em Fungos, Algas e Plantas, Universidade Federal de Santa Catarina Santa Catarina Brazil

**Keywords:** eDNA, next‐generation sequence, phyllosphere, rhizosphere, vindodden

## Abstract

*Dryas octopetala* is one of the most important botanical components of Arctic tundra. In parts of the Norwegian High Arctic Archipelago of Svalbard it can face strong grazing pressure, in particular of its flowers, by the Svalbard reindeer, whilst its production of mature viable seeds may be impacted by climate changes. Diverse organisms are associated with the habitat provided by flowering plants, some with the roots (rhizosphere) and others with the above‐ground surface of a plant (phyllosphere). Climatic changes affecting Svalbard may lead to the local expansion or reduction of plant populations and their associated communities. In this study, we carried out an initial investigation of non‐fungal eukaryotic communities associated with *D. octopetala* collected from four sampling locations at Vindodden on Svalbard using DNA metabarcoding. The diversity of organisms assigned based on the DNA sequences obtained was higher in the rhizosphere (6 phyla) than in the phyllosphere (11 phyla). The assignments included taxa that are common in Svalbard as well as some from various parts of the world but not recorded from the archipelago.

## Graphical abstract

- A single marker revealed a considerable diversity of organisms associate with *Dryas octopetala* .
- The rhizosphere contains more taxa than the phyllosphere.
- Some taxa are cited for the first time in the Svalbard Archipelago.
- The use of different DNA marker will increase diversity data in the Svalbard Archipelago.

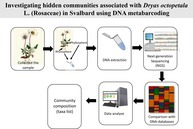

## 1 Introduction

The Arctic region currently faces considerable challenges from changing climate, in particular as it is warming two to four times more rapidly than the global average (Grigorieva 2024), the ‘Arctic amplification’ that is a fundamental aspect of Arctic climate change (Post et al. 2019; Przybylak et al. 2022; Rantanen et al. 2022; Polyakov et al. 2024). For example, in the summer of 2016, high Arctic latitudes faced extended periods of record‐breaking heat which continued late into the year, with temperatures during October–December, on average, 5°C above the norm (Simpkins 2017). In 2020, parts of northern Siberia reached an absolute record of 38°C north of the Arctic Circle (Overland and Wang 2021).

Arctic ecosystems and biodiversity are strongly influenced by climatic constraints (Callaghan et al. 2004). The generally low temperatures provide a barrier limiting the ability of arriving non‐native species to survive and establish in the region, a barrier that is likely to reduce as temperatures warm (Gilg et al. 2012). This challenge is likely to be amplified in synergy with growing anthropogenic activities in the region relating to trade, resource exploitation and tourism (Convey et al. 2012; Hall et al. 2010). Concurrent changes in precipitation, permafrost thaw, and reduction or change in ice and snow cover will create new conditions enabling some species—both native and exotic—to spread whilst others may shrink or even be lost (Gilg et al. 2012).

Many other organisms are associated with the habitat provided by flowering plants, whose distribution can be related to the distribution of the host plant. The term ‘rhizosphere’ was coined more than 100 years ago to refer to the soil surrounding and influenced by plant roots (Hiltner 1904), it is now widely appreciated that microorganisms inhabiting the rhizosphere can interact with and influence multiple aspects of the plant's biology (Lu et al. 2018). More recently, the term ‘phyllosphere’ has been adopted to describe the ‘total above‐ground surface of a plant when viewed as a habitat for microorganisms’ (Last 1955). The ecological relevance of the phyllosphere was further developed by Warren (2022). In all these habitats, most research attention to date has been given to bacteria and fungi living associated with the respective habitats (Aleklett et al. 2014; Bashir et al. 2022), but at present little is known about the presence or roles of other eukaryotic life forms such as protozoans, algae and ciliates.

Contemporary climate changes may lead to the expansion or reduction of plant populations (Chang et al. 2015; Reed et al. 2021) and, recognising that plants are associated with many other organisms, there is a need to better describe and understand the communities associated with the plant rhizosphere and phyllosphere that will also be exposed to the impacts of ongoing climate changes. At present, very limited data are available about such communities associated with Arctic flowering plants other than relating to fungi and bacteria (Bashir et al. 2022). Traditional approaches to investigating the diversity of these organisms are very challenging, requiring specialist taxonomic expertise across diverse groups of organisms, whilst community responses, including extinction, may now take place more rapidly than our ability to carry out surveys. Recent developments in molecular tools have allowed considerable and rapid advances in molecular diversity surveys in environmental samples. DNA metabarcoding by high‐throughput sequencing (HTS) is an efficient method for the detection of environmental DNA (eDNA) (Rippin et al. 2018; Ruppert et al. 2019; Câmara et al. 2022; Câmara, Carvalho‐Silva, et al. 2023), including that of rare species, spores and resting stages which are typically not detected in traditional morphology‐based surveys.

*Dryas* is a genus of Rosaceae and is a dominant dwarf shrub in the Arctic in terms of biomass (Billault‐Penneteau et al. 2019). One of the most widespread and common plant species present in the Arctic is *D. octopetala* L. (Rosaceae), known as the ‘mountain avens’. It is a small shrub with an Arctic‐Alpine distribution (Elkington 1965). It is one of the most important botanical biomass components of Arctic tundra (Skrede et al. 2006). Its name has been adopted for a period of Earth's geological history, the Younger Dryas (13,000–1700 years before present), which was characterised by a sudden drop in global temperature over the Northern Hemisphere, a period from which fossils of *Dryas* species are abundant (Mangerud et al. 2008). *Dryas octopetala* is typically a pioneer species in high pH (calcareous) areas and is very resistant to frost damage (Elkington 1965). It plays a role in nitrogen fixation, a process that occurs in its root nodules (Billault‐Penneteau et al. 2019). In the Norwegian High Arctic archipelago of Svalbard, it is known as ‘Reinrose’ and, having an almost ubiquitous distribution in Svalbard's Arctic tundra, is listed as Least Concern (LC) (https://svalbardflora.no/index.php/dryas/dryas‐octopetala). However, according to Cooper and Wookey (2003), the species is locally under strong grazing pressure, in particular its flowers, by the Svalbard reindeer, and its production of mature viable seeds is limited by the amount of thermal energy available during the short and cool Arctic summer growing season (Wookey et al. 1993, 1995; Naoya 1999). *Dryas octopetala* has been widely used as a model species in a range of ecological and evolutionary studies, including those focused in climate change (Skrede et al. 2006) and microbial community composition and dynamics (D'Alò et al. 2024). According to Kougioumoutzis et al. (2021) the species demonstrated resilience to the climatic fluctuations that occurred during the transition into the current interglacial period. However, projections suggest that, in the coming decades, the species may decline or become locally extinct due effects of climate‐change, increased interspecific competition, herbivory pressure, and the emergence or spread of plant pathogens.

In the present study, we investigated the diversity of non‐fungal eukaryotic organisms associated with the rhizosphere and phyllosphere of *D. octopetala* in Svalbard, employing the ITS2 molecular marker for DNA metabarcoding.

## 2 Materials and Methods

### 2.1 Sampling

**FIGURE 1:**
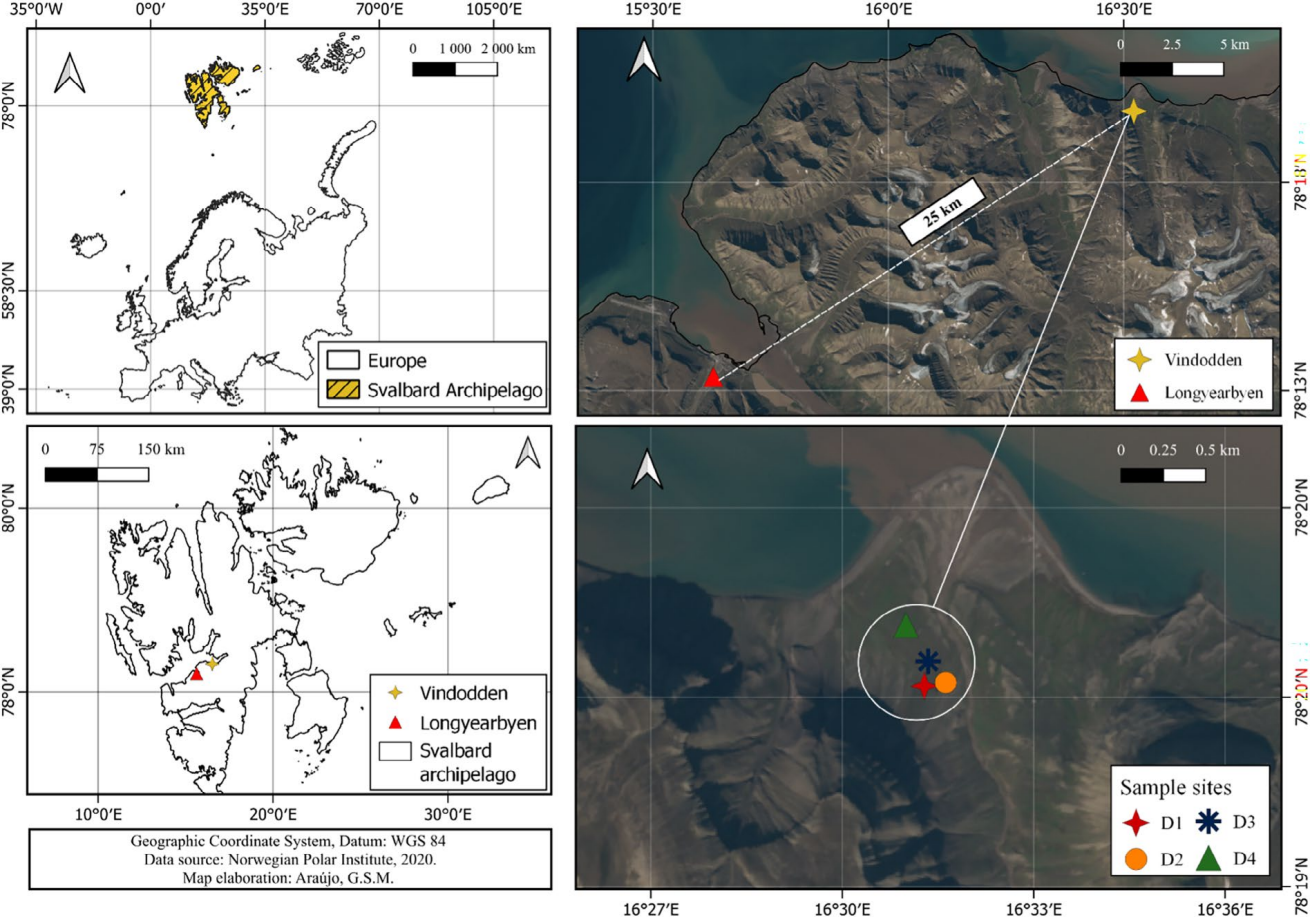
Map showing the sampling location in the Svalbard archipelago.

Samples were collected during fieldwork conducted in Svalbard between 10 and 14 July 2023. The High Arctic Svalbard archipelago is located in the Barents Sea at 78–80° N. Our study location, Vindodden, is located about 25 km from Longyearbyen, at 16° E, 70° N, from which it is accessible by boat in summer (Figure 1). At Vindodden, four specific sites were selected for sampling *D. octopetala* (numbered D1–D4), collecting the rhizosphere and phyllosphere (Table 1).

**TABLE 1:** Sampling locations.

Site	Lat/Long	Elevation	Rhizosphere	Phyllosphere
Site D1	N 78° 19.835′ E 016° 31.287′	41 m	Yes	No
Site D2	N 78° 19.871′ E 016° 31.619′	28 m	Yes	No
Site D3	N 78° 19.913′ E 016° 31.407′	35 m	Yes	Yes
Site D4	N 78° 20.023′ E 016° 30.999′	22 m	Yes	Yes

At each sampling site, triplicate samples (three individual plants) were collected separately. The roots were separated from the bulk soil using a tweezer to compose the rhizosphere. To sample the phyllosphere, stems and leaves were collected using scissors. Sterile gloves and tools were used, with material immediately sealed in sterile WhirlPack bags and immediately frozen (−20°C) on return to Longyearbyen (5 h) until DNA extractions were performed (within 2 days). Voucher specimens (Carvalho‐Silva et al. 2021) were deposited in the UB herbarium, University of Brasilia. All Dryas sequence assignments are assumed to refer to *D. octopetala* due to the uncertainties raised by Withe and Pirro.

### 2.2 DNA Extraction and Sequencing

Total DNA was extracted using the FastDNA Spin Kit for Soil (MPBIO, Ohio, USA), following the manufacturer's instructions. DNA quality was analyzed using agarose gel electrophoresis (1% agarose in 1 × Tris Borate‐EDTA) and then quantified using the Quant‐iT PicoGreen dsDNA Assay (Invitrogen). A negative control was included. For two of the four samples (D1, D2), the DNA yield from the phyllosphere was insufficient for extraction. The internal transcribed spacer 2 (ITS2) of the nuclear ribosomal DNA (Chen et al. 2010; Richardson et al. 2015; Câmara et al. 2022) was used as a barcode. PCR amplicons were generated using the primers specified by White et al. (1990) and were sequenced commercially using high throughput sequencing by Macrogen Inc. (South Korea) on an Illumina MiSeq sequencer (3 × 300 bp).

### 2.3 Data Analyses and Taxa Assignment

Quality analysis was carried out using BBDuk v. 38.87 in BBmap software with the following parameters: Illumina adapters removing (Illumina artefacts and the PhiX Control v3 Library); ktrim ¼ l; k ¼ 23; mink ¼ 11; hdist ¼ 1; minlen ¼ 50; tpe; tbo; qtrim ¼ rl; trimq ¼ 20; ftm ¼ 5; maq ¼ 20. Sequences remaining after quality control were imported to QIIME2 version 2023.9 (https://qiime2.org/) for bioinformatics analyses (Bolyen et al. 2019). The qiime2‐dada2 plugin was used for filtering, dereplication, turning paired‐end fastq files into merged and removing chimaeras, using default parameters (Callahan et al. 2016). Taxonomic assignments of amplicon sequence variants (ASVs) were determined using the qiime2‐feature‐classifier (Bokulich et al. 2018) classify‐sklearn against the curated databases PLANiTS2 and UNITE; the sequence similarity threshold used was 97%. For ITS2, firstly, ASVs were classified against the PLANiTS2 database (Banchi et al. 2020). After this step, ASVs that remained unclassified were filtered and classify‐sklearn classified against the UNITE Eukaryotes ITS database version 8.3 (Abarenkov et al. 2020). Finally, the remaining unclassified ASVs were filtered and aligned against the filtered NCBI non‐redundant nucleotide sequences (nt) database (May 2024) using BLASTn (Camacho et al. 2009) with default parameters; the nt database was filtered with the following keywords: ‘ITS1’, ‘ITS2’, ‘Internal transcribed spacer’, and ‘internal transcribed spacer’. Taxonomic assignments were performed using MEGAN6. For simplicity, we henceforth refer to the assigned ASVs as ‘taxa’. For comparative purposes, we consider reads as a proxy for relative abundance (Deiner et al. 2017; Hering et al. 2018; Câmara et al. 2022; Carvalho‐Silva et al. 2021). Rarefaction curves were generated using the software PAST 3.26 (Hammer et al. 2001). Venn diagrams were prepared as described by Heberle et al. (2015).

Information about distribution and habitat was obtained from GBIF (www.gbif.org), *AlgaeBase* (www.algaebase.org) and relevant studies in the literature.

## 3 Results

**FIGURE 2:**
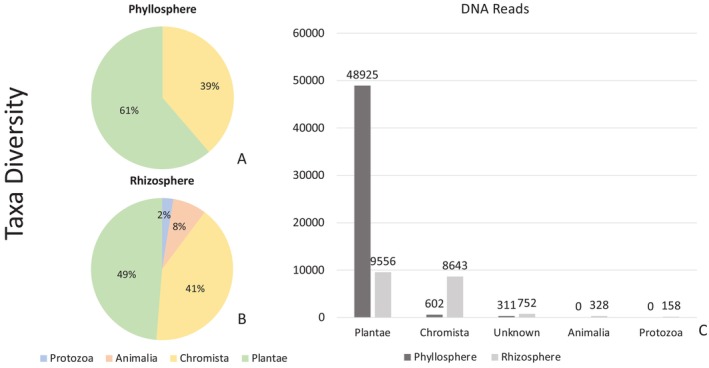
(A) Number of assigned taxa found in the *Dryas octopetala* phyllosphere. (B) Number of assigned taxa found in the *D. octopetala* rhizosphere. (C) Number of DNA reads by group in the rhizosphere and phyllosphere.

**FIGURE 3:**
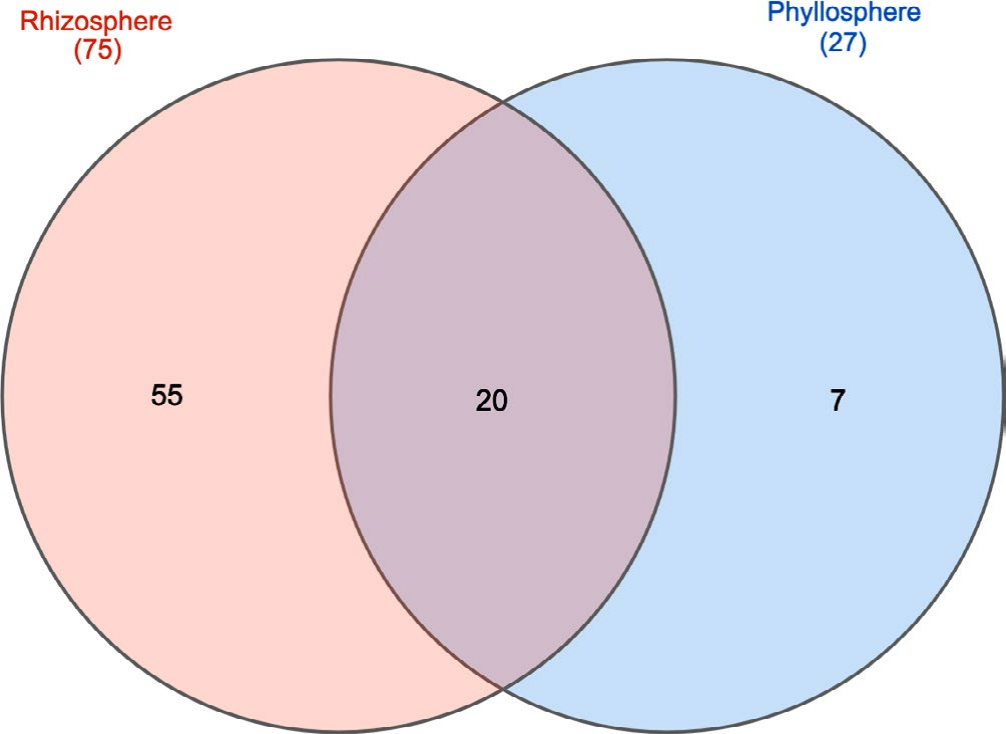
Venn diagram showing number of assigned taxa and their overlap in the *Dryas octopetala* phyllosphere and rhizosphere.

A total of 1,189,078 DNA reads remained after cleaning and denoising, of which 687,070 were assigned to the kingdom Fungi and will form the subject of a separate paper. A total of 502,008 DNA reads were assigned to non‐fungal eukaryotes, representing 85 ASVs (excluding the host plant *D. octopetala*). Samples from the phyllosphere (Table 2) included a total of 27 assigned taxa representing two kingdoms and five phyla. A total of 311 reads (ca. 0.07%) could not be assigned to any taxonomic rank (Figure 2). A total of 75 taxa were assigned from the rhizosphere samples, representing four kingdoms and 11 phyla (Table 3), with 752 DNA reads (ca. 0.4%) not being assigned to any taxonomic rank (Figure 2). Of the 85 assigned taxa, 20 were shared between the rhizosphere and phyllosphere, whilst only seven were restricted to the phyllosphere (Figure 3). About 86.3% (phyllosphere) and 93.09% (rhizosphere) of reads represented the host plant *D. octopetala* , and were not included in our analyses.

**TABLE 2:** Taxa present (and their global distribution) in the *Dryas octopetala* phyllosphere and numbers of DNA reads obtained from each sampling site.

Taxa	Distribution/habitat	DNA reads	
		Site D3	Site D4
KINGDOM CHROMISTA			
PHYLUM CERCOZOA	W/C	0	22
PHYLUM CILIOPHORA	W/C	9	13
Class Gymnostomatea			
*Parafurgasonia* sp.	Tr, STr/F, S	0	48
Class Oligohymenophorea	W/C	0	11
*Homalogastra* sp.	W/F	0	21
*Opisthonecta* sp.	Eu/Sw	6	0
*Vorticella* sp.	W/C	0	118
Class Spirotrichea			
*Schmidingerothrix* sp.	Af/S	0	8
Order Sporadotrichida	W/C	90	105
Fam. Oxytrichidae	W/C	91	0
*Cyrtohymena* sp.	W/F	25	0
*Sterkiella histriomuscorum*	W/F, S	0	35
KINGDOM PLANTAE			
PHYLUM ANTHOPHYTA			
Fam. Lamiaceae			
*Stachys acerosa*	Iran	0	394
Fam. Orobanchaceae			
*Pedicularis* sp.			27
*Bistorta vivipara*	Ar	0	57
Fam. Ranunculaceae			
*Ranunculus carpaticola*	Eurasia	0	22,280
Fam. Rosaceae			
*Dryas alaskensis*	Alaska	416	200
*Dryas octopetala*	Ar, Al	137,527	168,668
*Dryas* sp.	Ar, Al	648	458
PHYLUM BRYOPHYTA			
Fam. Pottiaceae	W/C	0	70
*Bryoerythrophyllum recurvirostrum*	W	0	38
*Syntrichia* sp.	W	255	0
Fam. Grimmiaceae			
*Racomitrium lanuginosum*	CP, Ad	21,837	0
Fam. Dicranaceae			
*Dicranoloma chilense*	Southern Chile	0	56
Fam. Plagiotheciaceae			
*Myurella* sp.	W	9	0
Fam. Brachytheciaceae			
*Brachythecium* sp.	W	0	914
PHYLUM CHLOROPHYTA	W/C	0	4
Fam. Stichococcaceae			
Stichococcus sp.		24	0
Fam. Trebouxiaceae			
*Trebouxia asymetrica*		648	458
Order Ulotrichales			
*Planophila* sp.		0	104
UNKNOWN		71	240

Abbreviations: A, Aerial; Ad, Andes; Af, Africa; Al, Alpine; An, Antarctica; Ar, Arctic; B, Brackish; C, Cosmopolitan; CP, Circumpolar; F, Freshwater; M, Marine; *P*, pooly known; S, Soil; Sar, Sub‐Arctic; STr, Sub‐tropical; Sw, Sewage; T, temperate; Tr, Tropical; W, Widespread.

**TABLE 3:** Taxa present (and their global distribution) in the *Dryas octopetala* rhizosphere and numbers of DNA reads obtained from each sampling site.

Taxa	Distribution/habitat	DNA reads			
		Site D1	Site D2	Site D3	Site D4
KINGDOM CHROMISTA					
PHYLUM BACILLARIOPHYTA	W/C	13	0	0	0
PHYLUM CERCOZOA	W/C	34	0	12	11
PHYLUM ENDOMYXA					
Fam. Plasmodiophoridae	W/C	0	0	0	2
PHYLUM CILIOPHORA	W/C	205	0	234	214
Class Gymnostomatea					
*Arcuospathidium* sp.	Eu, As, NA/F	0	0	14	0
Class Nassophorea					
*Parafurgasonia* sp.	Tr, STr/F, S	16	0	31	0
Class Oligohymenophorea	W/C	208	0	25	305
*Homalogastra* sp.	W/F	66	0	0	17
*Opisthonecta* sp.	Eu/Sw	0	0	0	32
*Rhabdostyla* sp.	W/F	0	0	0	45
*Zoothamnium* sp.	W/F, M	0	0	0	17
Fam. Stokesiidae	W/C	0	0	0	10
Fam. Vorticellidae		0	0	0	399
*Epicarchesium* sp.	W/F	7	0	0	0
*Vorticella* sp.	W/C	265	0	0	399
*Vorticellides* sp.	W/C	27	0	0	79
Class Oligotrichea					
*Strombidium* sp.	W/M	48	0	0	0
Class Spirotrichea	W/C	162	0	8	132
*Euplotes* sp.	W/C	5	0	0	0
*Schmidingerothrix* sp.	W/F	203	0	15	0
Order Sporadotrichida	W/C	1222	0	143	331
*Kahliella* sp.	W/F, M	328	0	0	47
Fam. Halteriidae	W/C	8	0	0	0
*Halteria* sp.	W/F, M	37	0	0	0
Fam. Oxytrichidae	W/C	725	0	204	324
*Cyrtohymena* sp.	W/F	105	0	0	0
*Sterkiella histriomuscorum*	W/F, Sw	127	0	0	29
*Stylonychia* sp.	W/F, M	11	0	0	0
Order Urostylida	W/C	1266	0	0	19
*Anteholosticha* sp.	W/F	111	0	0	40
*Hemicycliostyla* sp.	W/F	0	0	0	9
*Uroleptus* sp.	W/F	297	0	0	0
KINGDOM PROTOZOA					11
PHYLUM EVOSEA	W/C	33	0	75	0
PHYLUM SULCOZOA					
Fam. Apuzominadidae	W/F	39	0	0	0
KINGDOM ANIMALIA					
PHYLUM NEMATODA					
*Acrobeles* sp.	W/T	3	0	0	0
*Acrobeloides* sp.	W/T	0	0	44	0
PHYLUM ARTHROPODA	W/C	16	0	0	0
Subclass Collembola					
*Folsomia quadrioculata*	W/T	0	0	0	46
*Folsomia* sp.	W/T	0	0	203	0
*Protaphorura* sp.	W/T	16	0	0	0
KINGDOM PLANTAE					
PHYLUM ANTHOPHYTA					
Fam. Caryophyllaceae					
*Cerastium velutinum*	NA	0	0	41	0
Fam. Caryophyllaceae					
*Sabulina rubella*		19	0	0	0
Fam. Lamiaceae					
*Stachys acerosa*	Iran	1166	89	0	512
Fam. Orobanchaceae					
*Pedicularis* sp.	Ar, SArc, Temp	0	0	0	0
Fam. Polygonaceae					
*Bistorta vivipara*	Ar	0	0	0	50
Fam. Rosaceae					
*Dryas alaskensis*	Alaska	0	154	0	0
*Dryas octopetala*	Ar, Al	15,238	82,113	304	8594
*Dryas* sp.	Ar, Al	0	75	15,966	4276
Fam. Salicaceae					
*Salix* sp.	W	8	0	0	0
Fam. Saxifragaceae					
*Saxifraga* sp.	T, Ar	105	0	0	0
PHYLUM BRYOPHYTA					
Fam. Bryaceae					
*Ptychostomum lonchocaulon*	NA	555	0	0	144
Fam. Dicranaceae	W	27	0	49	0
Fam. Ditrichaceae					
*Distichium* sp.	W	129	0	0	19
Fam. Encalyptaceae					
*Encalypta* sp.	W	31	0	0	0
Fam. Fissidentaceae					
*Fissidens* sp.	W	38	0	0	0
Fam. Grimmiaceae					
*Racomitrium lanuginosum*	CP, Ad	85	0	0	0
Fam. Orthotrichaceae					
*Amphidium* sp.	W	0	0	0	39
Fam. Plagiotheciaceae					
*Myurella* sp.	W	0	0	23	23
Fam. Pottiaceae		0	0	0	0
*Bryoerythrophyllum recurvirostrum*	W	208	0	35	0
*Didymodon tomaculosus*	EU, As	54	0	0	0
*Didymodon* sp.	W	1913	0	173	0
*Pterygoneurum ovatum*	W	8	0	0	0
*Syntrichia* sp.	W	356	0	0	0
*Tortula* sp.	W	114	0	31	0
Fam. Pylaisiaceae					
*Roaldia revoluta*	BP	51	0	0	0
PHYLUM CHLOROPHYTA	W/C	17	0	0	0
Class Trebouxiophyceae	W/C	59	0	0	0
Order Prasiolales					
*Diplosphaera* sp.	W/F, M	32	0	0	0
*Desmococcus spinocystis*	W/F, T	74	0	0	0
*Stichococcus bacillaris*	W/C	15	0	0	0
Order Trebouxiales					
*Coccomyxa* sp.	W/F, M	28	0	0	0
Order Trebouxiales					
*Dictyochloropsis* sp.	W/F, M	10	0	0	0
*Myrmecia pyriformis*	Ar, Eu, NA, SEA/F	139	0	0	0
*Myrmecia bisecta*	W/F	41	0	0	0
*Trebouxia asymmetrica*	Eu/F, A	75	154	0	0
*Trebouxia flava*	W/F, A, T	0	0	0	11
Order Ulotrichales					
*Planophila* sp.	Eu, NA/M	51	0	2601	0
UNKNOWN		556	0	124	72

Abbreviations: Al, Alpine; Ar, Arctic; As, Asia; BP, Bipolar; C, Cosmopolitan; CB, Circumboreal; F, Freshwater; M, Marine; NA, North America; *P*, Poorly known; SEA, South‐East Asia; STr, Sub‐tropical; SubA, Sub‐Antarctic; T, Temperate; Tr, Tropical; W, Widespread.

**FIGURE 4:**
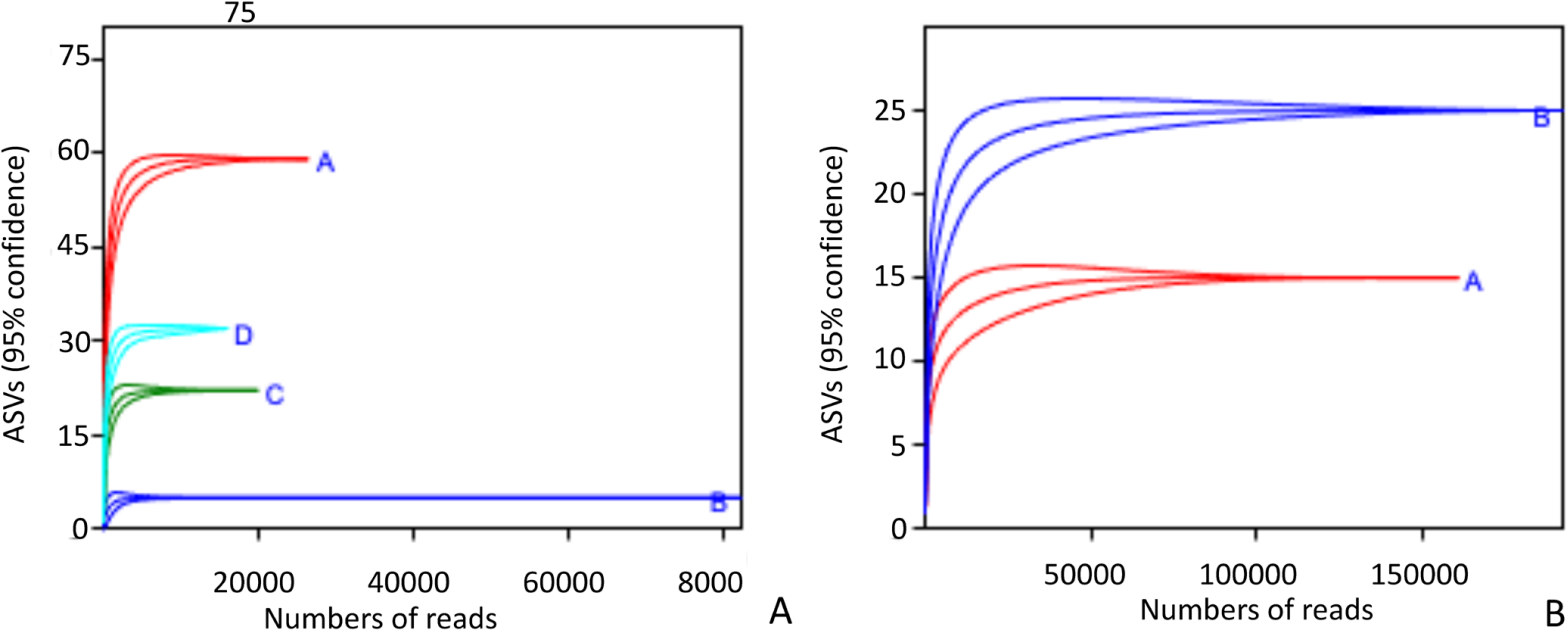
Rarefaction curves obtained from each sampling site. (A) Rhizosphere, A = Site 1, B = Site 2, C = Site 3, D = Site 4. (B) Phyllosphere, A = Site3, B = Site 4.

Rarefaction curves all reached a plateau (Figure 4), indicating that the sequencing depth sufficiently captured the diversity of the sampled communities adequately.

## 4 Discussion

The communities associated with *D. octopetala* in High Arctic Svalbard, as identified through DNA metabarcoding, encompass 11 phyla based on data generated using a single marker (ITS2). These findings highlight the high diversity associated with the roots, stems and leaves (rhizosphere and phyllosphere) of this common and ecologically important species in Svalbard.

Our data indicated that the plant rhizosphere hosted a higher diversity of taxa, representing a larger number of phyla, than did the phyllosphere. For many organisms, the sub‐surface habitat provides higher humidity, more thermal stability and protection from wind abrasion and solar radiation. Many typical soil organisms (e.g., nematodes, springtails and some ciliates) are more diverse in the more sheltered root‐associated environment. The presence of marine taxa amongst the ASV assignments is likely a reflection of the proximity of the sampling locations to the shoreline (< 100 m), representing wind‐blown biological material from the marine environment.

The Kingdom Plantae contributed the vast majority, 61% and 49%, respectively, of the number of non‐fungal reads from the phyllosphere and rhizosphere, even after exclusion of the dominant assignment to the host plant species. The Phylum Anthophyta was represented in both phyllosphere and rhizosphere, by six and nine taxa, respectively. Almost all of the genera assigned are known to occur in the Arctic, including on Svalbard, with the exception of *Stachys acerosa*, which is a species native to Iran and widely employed in conventional healthcare in various countries.

*Ranunculus carpaticola* represented 45% of the sequences assigned to the phylum Plantae. The species is not native to Svalbard, but at least three other species of the genus *Ranunculus* are common in the region. It is important to note that the sequenced material may originate from minute plant fragments containing DNA, such as pollen grains or other propagules, whilst all eDNA studies are subject to the limitations of sequences deposited in the available databases. Furthermore, although Svalbard is a remote archipelago with geographic barriers to natural dispersal, 98 non‐native plant species, including representatives of *Ranunculus*, have been documented (Bartlett et al. 2021).

Representatives of the phylum Bryophyta included 15 taxa, with five assigned from the phyllosphere and 13 from the rhizosphere. However, only the species *Racomitrium lanuginosum* , a common Svalbard native (Wietrzyk‐Pełka et al. 2020), was notably abundant in the phyllosphere. *Bryoerythrophyllum recurvirostrum* is also native to Svalbard, but provided a low number of reads. The other assigned species are known to occur elsewhere in the Arctic, but not in Svalbard, with the exception of *Dicranoloma chilense* which is known only from southern Chile (Ireland et al. 2010). The latter is again most likely an illustration of the limitations of the available databases. The phylum Chorophyta was less strongly represented, with 12 species assigned in the rhizosphere samples and four in the phyllosphere. The majority of these are widespread and common taxa, whilst some (e.g., *Trebouxia flava*, *Stichococcus bacillaris* ) are known lichen photobionts (Thüs et al. 2021).

The high proportion of plant assignments in our samples is expected, given the relatively diverse local flora (Ingebrigtsen et al. 2017; Wietrzyk‐Pełka et al. 2020) and the timing of sample collection in the Arctic summer, when the plants were actively producing flowers, fruit or other propagules. Additionally, the morphological characteristics of *D. octopetala, with* short, tomentose stems and densely pubescent leaves, will facilitate the capture and retention of external material, potentially contributing to the accumulation of DNA from other plant species on its surface.

Representatives of the Kingdom Chromista were also more diverse in the rhizosphere with 26 assigned taxa, against 12 from the phyllosphere. The majority are widespread free‐living organisms. However, the assignment of *Schmidingerothrix* is surprising, as the genus only has one known species (*S. extraordinaria*) recorded only from saline soils in Egypt and Namibia (Foissner 2012). Although low numbers of DNA reads were obtained (< 1%, 220 reads), this assignment was made from both rhizosphere and phyllosphere.

The presence of non‐native taxa amongst our assignments is not necessarily unexpected as, even if not viable or capable of establishment, such taxa may be transported in the air column, via zoochory associated with migratory birds, or with anthropogenic assistance, even in the polar regions. As noted above, a wide range of non‐native plant taxa has been reported on Svalbard (Bartlett et al. 2021). Câmara et al. (2022), analysing air samples obtained across a 40° latitude transect from South America to Antarctica, suggested that non‐native biota or propagules may be transported such distances, whilst aerobiological studies and molecular phylogenetic analyses have confirmed such transport or linkages at even bipolar scale for various microbiota and mosses (Pearce et al. 2017; Kleinteich et al. 2017; Biersma et al. 2017). Svalbard can be considered a ‘hotspot’ for non‐native species, in part due to association with anthropogenically imported or influenced soils enriched with nutrients (Liska and Soldán 2004; Coulson et al. 2024). Ware et al. (2022) sampled the footwear of 259 travellers arriving in Svalbard in 2008, and found 1019 plant seeds and 465 bryophyte fragments.

The internal transcribed spacer 2 (ITS2) region of the nuclear ribosomal DNA has been widely used to identify fungi (Tedersoo et al. 2022; Gonçalves et al. 2025) as well as a broad range of other eukaryotic organisms, including animals, protozoans, chromists and plants (Ruppert et al. 2019; Carvalho‐Silva et al. 2021). Taxonomic assignment using ITS2 is supported by well‐established reference databases such as UNITE for fungal sequences and GenBank for general eukaryotic sequences. These resources have proven effective in facilitating the identification of environmental DNA (eDNA), and have been successfully applied in recent studies of bryosphere diversity conducted by our group (Câmara et al. 2022, 2024; Câmara, Lopes, et al. 2023).

It is important to note that there is no single universal genetic marker suitable for all eukaryotic microorganisms. Each marker presents advantages and limitations depending on the taxonomic group of interest. Amongst available markers, the 18S rRNA gene is the most widely used for eukaryotic metabarcoding studies (e.g., Pushkareva et al. 2022). However, 18S often lacks sufficient taxonomic resolution, particularly at the species level, and is thus suboptimal for distinguishing closely related taxa, especially amongst microalgae and plants (Lara et al. 2022). For instance, although Pushkareva et al. (2022) analyzed soil samples from Svalbard using the 18S rRNA marker, and reported a high number of eukaryotic ASVs, the taxonomic resolution in that study was limited to higher taxonomic levels (Kingdom and Phylum), making it impossible to make comparisons with our study. Studies with phyllosphere in the Arctic region often include bacterial and fungal microbiota (Sohrabi et al. 2023; Son and Lee 2022).

The symbiotic relationships between *D. octopetala* and fungal and bacterial communities are frequently noted (Bjorbækmo et al. 2010; D'Alò et al. 2024; Son and Lee 2022), but to other organisms the literature is limited.

Most of the taxa detected in association with *D. octopetala* in this study represent environmental contaminants, with 84.4% of the sequences identified being assigned to the Kingdom Plantae. This may indicate a methodological bias, given that the ITS2 marker is highly sensitive in detecting plants and microalgae DNA, often enabling taxonomic resolution at the species level (Heeger et al. 2019).

Most of the assigned taxa from the phyllosphere plausibly derive from fragments of plant tissue, generally corresponding to the known local flora. Other organisms, such as *Cyrtohymena* sp., *Sterkiella histriomuscorum, Arcuospathidium* and *Opisthonecta* are frequently associated with biofilms on roots or other plant surfaces These ciliates do not form symbiotic relationships with the plants, but may still confer indirect benefits. For instance, through predation on bacteria or fungi, these ciliates could contribute to the regulation of microbial communities, potentially reducing the abundance of phytopathogenic or competitive bacterial taxa.

The detection of soil‐dwelling taxa (*Acrobeles* sp., *Acrobeloides* sp., *Folsomia quadrioculata* ) indicates the presence of organisms involved in decomposition processes. These taxa play critical roles in nutrient recycling and soil ecosystem functioning by facilitating the breakdown of organic matter and enhancing nutrient availability, thereby contributing to overall soil health.

We recognise that DNA metabarcoding studies are highly dependent on the quality and completeness of available databases, whilst sequence assignments do not confirm the presence of a living or viable organism. The production of DNA reads that could not be taxonomically assigned may indicate the limitations of available databases, or could represent currently undescribed and non‐sequenced taxa.

## 5 Conclusions

The use of a single marker (ITS2) in this metabarcoding study revealed a diversity of organisms present in association with *D. octopetala* . Whilst most assigned taxa are known from Svalbard or other Arctic regions, a small proportion of assignments were to taxa with very restricted distributions far from the polar regions. We accept that the occurrence of these species in Svalbard is highly unlikely, and may illustrate the limitations of the methodology or of currently available databases. The use of different DNA markers (e.g., 16S, 18S or Cox1) in future studies will increase diversity data associated with the plant rhizosphere and phyllosphere. With ongoing rapid climatic change in the Arctic, the use of eDNA metabarcoding studies could provide a rapid and valuable tool for monitoring changes in vegetation‐associated diversity.

## Author Contributions

M.C.‐S., L.H.R., V.N.G., M.H.S.R., K.B.S., G.S.M.A., F.A.C.L., P.C. and P.E.A.S.C. conceived the study, analyzed the results and wrote the manuscript. M.C.‐S., L.H.R., V.N.G., M.H.S.R., P.C. and P.E.A.S.C. collected the samples. M.C.‐S., L.H.R., V.N.G., P.E.A.S.C. performed DNA extractions. F.A.C.L. performed the metabarcoding analysis. All authors read and approved the final manuscript.

## Conflicts of Interest

The authors declare no conflicts of interest.

## Data Availability

The NCBI database was used to store all of the raw data. The accession numbers are SAMN52459372, SAMN52459373, SAMN52459374, SAMN52459375, SAMN52459376, SAMN52459377, SAMN52459378, SAMN52459379, SAMN52459380, SAMN52459381, SAMN52459382, SAMN52459383.
